# Hsa_circ_0007099 and PIP4K2A coexpressed in diffuse large B-cell lymphoma with clinical significance

**DOI:** 10.1016/j.gendis.2023.06.025

**Published:** 2023-07-31

**Authors:** Jinghan Wang, Xin Ku, Qiuling Ma, Haikuo Li, Sujuan Huang, Liping Mao, Fang Yu, Jie Jin, Wei Yan

**Affiliations:** a Department of Hematology, The First Affiliated Hospital, Zhejiang University School of Medicine, Hangzhou, Zhejiang 310003, China; b Key Laboratory of Hematologic Malignancies, Diagnosis and Treatment, Hangzhou, Zhejiang 310003, China; c Zhejiang Provincial Clinical Research Center for Hematologic Diseases, Hangzhou, Zhejiang 310003, China; d Cancer Center, Zhejiang University, Hangzhou, Zhejiang 310003, China; e Shanghai Center for Systems Biomedicine, Key Laboratory of Systems Biomedicine (Ministry of Education), Shanghai Jiao Tong University, Shanghai 200240, China; f The Second School of Clinical Medicine, Henan University of Chinese Medicine, Zhengzhou, Henan 450046, China; g Department of Pathology, The First Affiliated Hospital, Zhejiang University School of Medicine, Hangzhou, Zhejiang 310003, China

Diffuse large B-cell lymphoma (DLBCL) is the most common subtype of B-cell lymphoma in adult patients. Due to the clinical and molecular heterogeneity of DLBCL patients, robust biomarkers in clinical practice are still required. Clinically, the international prognostic index (IPI) was considered the most well-established predictor. Molecularly, mRNA expression and genetic subtypes were regarded as useful biomarkers. However, the prognostic potential of circRNA expression in DLBCL patients is still unclear. CircRNAs are more stable than linear mRNAs. Due to their richness, stability, and tissue specificity, circRNAs should have a potential utility as cell-free biomarkers.^1^^,^^2^ Notably, B-cells have specific circRNA markers compared with T-cells. Additionally, circRNA expression profiles can distinguish different B-cell malignancies.^3^ Besides, circRNAs are expressed in higher amounts in some diseases than their corresponding linear mRNAs. Based on these studies, we used RNA sequencing to search for circRNAs related to disease progression and identified hsa_circ_0007099 as one of the significant predictors. Furthermore, the regulatory pathway of hsa_circ_0007099 was constructed in *in silico* analysis and validated in *in vitro* cellular experiments.

This study was conducted using a discovery and validation design (Table S1). First, we enrolled 17 pairs of DLBCL patients with progressed or relapsed disease within 24 months (POD24) and non-POD24 by matching for clinical and molecular factors such as age, Hans classification, double expressor lymphoma (DEL), and IPI (Fig. S1). After controlling these potential confounders, we identified 11 circular RNAs with clinical significance (Fig. 1A). Specifically, hsa_circ_0007099 and hsa_circ_0060,158 were positively associated with POD24. In contrast, hsa_circ_0098,305, hsa_circ_0115,422, hsa_circ_0004087, hsa_circ_0001529, hsa_circ_0004243, hsa_circ_0002004, hsa_circ_0003221, hsa_circ_0009128, and hsa_circ_0005535 were negatively associated with POD24 (Fig. S2). Since hsa_circ_0007099 is one of the most significant factors closely correlated with POD24, it was selected to investigate further. Hsa_circ_0007099 is derived from exons 6 and 7 of the abhydrolase domain containing 2, acylglycerol lipase (ABHD2, NM_007011.8), located on chr15 (q26.1). The sequence of the splice site was illustrated in Figure 1B. Notably, there was no association between *ABHD2* mRNA expression and overall survival in GSE57611, GSE32918, GSE21846, and TCGA datasets (Fig. S3). However, receiver operating characteristic (ROC) curves demonstrated that hsa_circ_0007099 expression was a potential predictor for POD24 in the training set of 34 DLBCL patients (the area under the curve/AUC = 0.97; Fig. S4). In addition, we estimated the sample size using a One ROC Curve Power Analysis and enrolled 82 patients with available formalin-fixed paraffin-embedded tissues as an independently validated cohort (Fig. S5). POD24 patients in this validated set were more common in males (*P* = 0.042), higher levels of LDH (*P* = 0.007), extranodal disease (*P* = 0.008), non-GCB subtype (*P* = 0.056), DEL (*P* = 0.016), and higher IPI scores (*P* = 0.004) (Table S1). As expected, both progression-free survival (PFS) and overall survival (OS) curves showed 30 (39%) POD24 patients had poor outcomes compared with 52 (61%) non-POD24 patients (Fig. S6). In multivariable logistic regression analysis, we found hsa_circ_0007099 expression as a continuous variable was positively associated with POD24 in the context of sex, Hans classification, DEL, and IPI (Fig. 1C). Next, we divided these patients into high and low expression groups according to the optional cutoff value of this circRNA expression by ROC curves (Fig. 1D). As a result, the AUC value was 0.79, confirming that hsa_circ_0007099 expression had an acceptable predictive power for POD24. Clinical characteristics of patients with high expressed hsa_circ_0007099 are summarized in Table S2. The proportion of male patients was higher in the high expression group than those in the low expression group (70.3% *vs*. 42.2%, *P* = 0.015). There was no statistical correlation between hsa_circ_0007099 expression and other variables including age, LDH, poor ECOG performance status, advanced disease (Stage III–IV), extranodal involvements, IPI, Hans classification, and DEL. The low expressers (*n* = 45) had longer PFS (Fig. 1E) and OS (Fig. S7) than the high expressers (*n* = 37). Furthermore, when hsa_circ_0007099 expression was used as a continuous variable or a categorical variable, its expression was consistently associated with poor PFS and OS, respectively (Table S3). In order to exclude the potential confounders, a multivariate analysis was performed (Table S4, 5). After adjusting for gender, IPI, Hans classification, and DEL, hsa_circ_0007099 expression was still an independent prognostic factor for PFS [HR (95% CI), 3.968 (1.903, 8.275), *P* < 0.001 for the categorical variable; HR (95% CI), 1.16 (1.01, 1.333), *P* = 0.036 for the continuous variable] and OS [HR (95% CI), 3.836 (1.779, 8.275), *P* = 0.001 for the categorical variable; HR (95% CI), 1.18 (1.011, 1.378), *P* = 0.036 for the continuous variable].

**Figure 1:**
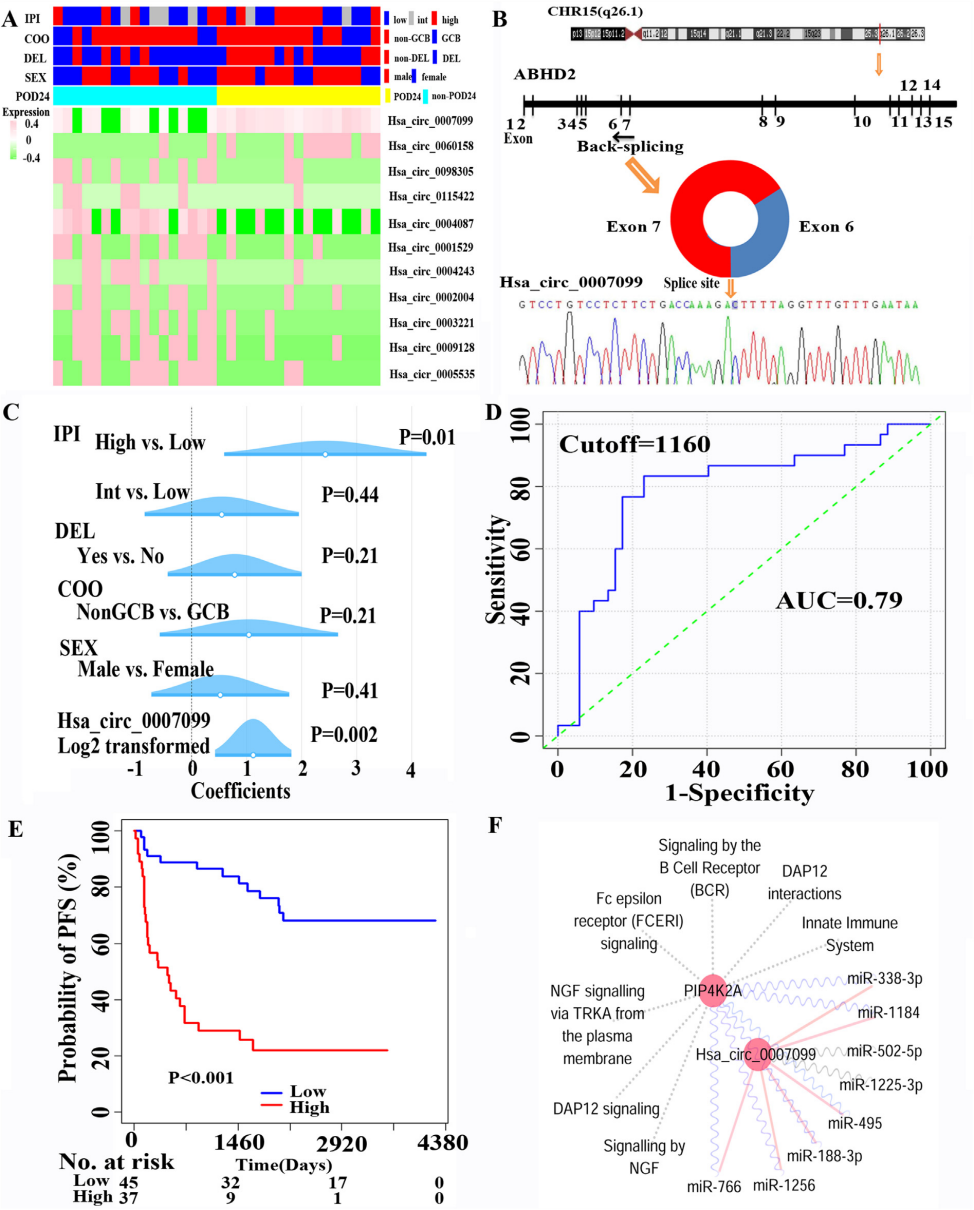
Differently expressed circRNAs related to POD24 were studied in 34 matched DLBCL patients, and hsa_circ_0007099 was validated as one of the significant predictors in an independent cohort of 82 patients. The regulatory networks involved in hsa_circ_0007099 were analyzed. **(A)** A total of 11 differently expressed circRNAs were illustrated in the heatmap. **(B)** The schematic illustration showed the circularization of *ABHD2* exon 6 to 7 formed hsa_circ_0007099 and the splice site was sequenced. **(C)** Multivariate logistic regression analysis was used to validate the independent predictive ability of hsa_circ_0007099 expression for POD24 in the validation set. **(D)** ROC curves were used to classify patients into high and low expression groups based on the optional cutoff value of this circRNA expression. **(E)** Survival curves of PFS for DLBCL patients of the high and low expression groups. **(F)** Has_circ_0007099 sponges eight miRNAs and then up-regulates the expression of *PIP4K2A* involving seven pathways.

In order to understand the biological insights of hsa_circ_0007099 overexpression, the protein expression profiles of DLBCL tissues were compared between six paired high and low expression groups. The clinical characteristics of these patients are summarized in Table S6. As a result, 25 proteins were up-regulated and 13 down-regulated in high expressers (Fig. S8). In the Reactome pathway analysis, these encoding genes were involved in 18 different Reactome pathways (Table S7). Notably, *PIP4K2A* was involved in seven pathways such as Fc epsilon receptor signaling, signaling by the B cell receptor, DAP12 interactions, DAP12 signaling, signaling by NGF, NGF signaling via TRKA from the plasma membrane, and innate immune system pathways (Fig. 1F). Additionally, high expression of *PIP4K2A* was positively correlated with poor OS, implying its oncogene role in DLBCLs (Fig. S9).

As previously reported, hsa_circ_0007099 was highly expressed in cervical cancer cells, breast cancer cells, lung cancer cells, and colon cancer tissues.^4^^,^^5^ Based on previous studies, hsa_circ_0007099 might play an oncogenic role in tumor development. Studies have shown that the most concerned mechanism of circRNA is to regulate the gene expression network by spongy adsorption of microRNAs. Here, we identified eight miRNAs that may interact with hsa_circ_0007099 in the CircInteractome database (Fig. 1F; Fig. S10). By miRNA-mRNA integrative analysis, we found 6 miRNAs targeted 12 genes (encoding aberrantly expressed proteins in the high expression group) in the miRNA-mRNA network (Fig. S11 and Table S8). Notably, the 3′UTR of *PIP4K2A* is predicted to bind to miRNAs of miR-188-3p, miR-1256, miR-1184, miR-338-3p, miR-495-3p, miR-495-5p genes. At the same time, the expression of *PIP4K2A* and hsa_circ_0007099 was positively correlated in 24 patients (Fig. S12). Thus, we speculated that hsa_circ_0007099 might up-regulate the expression of *PIP4K2A* via sequestering these 6 miRNAs, involving at least seven Reactome pathways. Therefore, we conducted cellular experiments to further validate this regulatory network. Knockdown expression of hsa_circ_0007099 was confirmed by Q-PCR analysis after 48-h shRNA treatment in OCI-Ly1 and OCI-Ly10 cell lines (Fig. S13). The knockdown of hsa_circ_0007099 expression significantly increased expression of miR-188-3p, miR-1256, miR-1184, miR-338-3p, miR-495-3p, miR-495-5p genes. In parallel, the knockdown of hsa_circ_0007099 expression significantly reduced the expression of *PIP4K2A.*

In summary, we found hsa_circ_0007099 could sponge 6 miRNAs and then up-regulate the expression of phosphatidylinositol-5-phosphate 4-kinase type 2 lipid kinase A (PIP4K2A) gene. Phosphatidylinositol-5,4-bisphosphate is the precursor to second messengers of the phosphoinositide signal transduction pathways, which is involved in the regulation of secretion, cell proliferation, differentiation, and motility. Thus, PIP4K2A in leukemia and solid cancers has attracted extensive attention due to its role in signal transduction, metabolic homeostasis, and genomic instability. Consistently, we found that *PIP4K2A* expression was associated with adverse OS (Fig. S9), suggesting an oncogenic role in lymphoma. Meanwhile, PIP4K2A was involved in seven pathways related to has_circ_0007099 overexpression (Fig. 1F).

There are still some limitations. Firstly, prospective validation in multicenter patients is still needed to validate this result. Secondly, further validation of the regulated axis of has_circ_0007099 using luciferase reporter assay, RNA pull-down assay, fluorescence *in situ* hybridization, RNA immunoprecipitation, and RNase protection assay are required.

In conclusion, hsa_circ_0007099 expression may independently contribute to the poor prognosis of DLBCL patients and present a novel therapeutic target.

## Conflict of interests

The authors declare that they have no competing interests.

## Funding

This work is supported by the 10.13039/501100001809National Natural Science Foundation of China (No. 81820108004), Interdisciplinary Program of Shanghai Jiao Tong University (YG2021QN46, YG2021QN126), Key Laboratory of Systems Biomedicine (Ministry of Education, KLSB2019KF-02), and the Institutional Review Boards of the First Affiliated Hospital of Zhejiang University (No. 2021769). The funders had no role in the study design, data collection, data analysis, interpretation, or writing of this report.
