# Supplementary material for: Hsa_circ_0007099 and PIP4K2A coexpressed in diffuse large B-cell lymphoma with clinical significance
Source: Genes Dis. 2023 Jul 31;11(4):101056. doi: 10.1016/j.gendis.2023.06.025 (PMC10950802; doi:10.1016/j.gendis.2023.06.025)
Supplement: Multimedia component 1 [file mmc1.docx]

**Supplementary methods**

**Methods**

**Patients**

The clinical data of DLBCL patients were obtained from the medical records of the First Affiliated Hospital of Zhejiang University. We included patients who were treated with R-CHOP (Rituximab 375 mg/m2 on Day 0, Cyclophosphamide 750 mg/m2, Doxorubicin 50 mg/m2, and Vincristine 1.4 mg/m2 intravenously on Day 1, and Predisone 60 mg/m2 orally on Days 1-5) chemotherapy. All cases were confirmed by pathological diagnoses and immunohistochemical staining. Patients who had previous malignancy and transformed from indolent lymphoma, double hit lymphoma, and HIV-associated DLBCLs were excluded. A total of 56 fresh frozen lymphoma tissues containing adequate material for RNA extraction were stored in our biobank. Of these samples, 17 patients who progressed or relapsed within 24 months (POD24) of initial therapy were assigned to the unfavorable group. To exclude potential confounders, we conducted a propensity score analysis based on age, gender, Hans classifications, DEL and IPI, selecting another 17 samples from non-POD24 patients as the favorable group (Figure S1). Therefore, a total of 34 patients were selected for circular RNA sequencing. The validated group consisted of 82 DLBCL cases obtained from paraffin wax DLBCL samples. The study was approved by the Institutional Review Boards of the First Affiliated Hospital of Zhejiang University (Reference Number 2021769).

**Quantitative reverse transcriptase-PCR**

Real-time PCR (RT-PCR) was used for relative quantification of circRNA using RNA from formalin fixed and paraffin-embedded (FFPE) tissues. Details of the RNA extraction method are described in the supplementary method. RNA was synthesized into cDNAs using the PrimeScriptTM RT reagent kit (TakaRa) according to manufacturer’s instructions. Quantitative RT-PCR was performed using 2× Taq PCR mix. The primers are listed as follows: hsa_circ_0007099 F: 5’- AGCCCCACCTGACCTCTACT -3’; hsa_circ_0007099 R: 5’- TCTTCCCACTGCAAATACGA -3’; beta-actin F: 5’- CATGTACGTTGCTATCCAGGC -3’; beta-actin R: 5’- CTCCTTAATGTCACGCACGAT -3’.

**Plasmid construction and lentiviral transduction**

Three short hairpin RNAs (shRNAs) targeting hsa_circ_0007099 (targeting sequence: TCTTCTGACCAAAGACTTT) and negative controls were designed and cloned into the lentiviral vector pGMLV-SC5 RNAi by Genomeditech. The above vectors were packaged into lentiviruses using psPAX2 and pMD2.G vectors, which were co-transfected in 293T cells using a calcium phosphate cell transfection kit (Beyotime Biotechnology, China) according to the manufacturer’s guidelines. Finally, the lentiviruses were transformed into lymphoma cells, and cells were then selected with puromycin (InvivoGen, USA). miRNA primers were listed in the following. miR-188-3p: CTCCCACATGCAGGGTTTGCA,

miR-338-3p: TCCAGCATCAGTGATTTTGTTG, miR-495-3p: AAACAAACATGGTGCACTTCTT, miR-766-3p: ACTCCAGCCCCACAGCCTCAGC,

miR-1184: CCTGCAGCGACTTGATGGCTTCC, miR-1256: AGGCATTGACTTCTCACTAGCT; circ-actin-F：CATGTACGTTGCTATCCAGGC;

circ-actin-R：CTCCTTAATGTCACGCACGAT. The sequences of shRNAs cloned into the lentiviral vector were shown in the following figure.


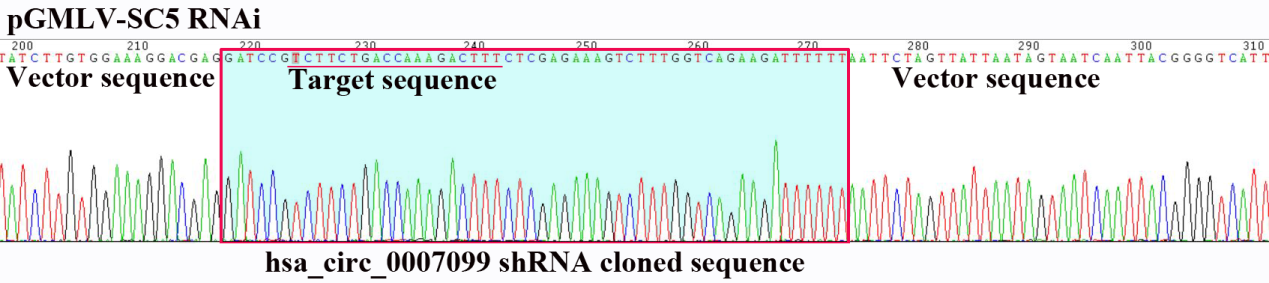


The sequences of shRNAs cloned into the lentiviral vector.

**Circular RNA sequencing analysis**

Total RNA from the fresh frozen samples was isolated by Trizol reagent (Invitrogen life technologies). RNA quality was evaluated using a Nanodrop ND-1000 (Thermo Fisher Scientific, Waltham, MA, USA). Transcriptome high throughput sequencing was done by Cloud-Seq Biotech (Shanghai, China). Briefly, total RNA was used for removing the rRNAs using Ribo-Zero rRNA Removal Kits (Illumina, USA) following the manufacturer's instructions. RNA libraries were constructed by using rRNA-depleted RNAs with TruSeq Stranded Total RNA Library Prep Kit (Illumina, USA) according to the manufacturer’s instructions. Libraries were controlled for quality and quantified using the BioAnalyzer 2100 system (Agilent Technologies, USA). Paired-end reads were harvested from Illumina HiSeq 4000 sequencer, and were quality controlled by Q30. After 3’ adaptor-trimming and low quality reads removing by "cutadapt" software (v1.9.3). The high quality trimmed reads were used to circRNAs analyses. These high quality reads were aligned to the reference transcriptome with "STAR" software and circRNAs were detected and identified with "DCC" software.

**RNA extraction for FFPE tissue samples**

In brief, up to five unstained slides (10 μm thickness) were generated from each FFPE block. The RNA extraction was performed by AmoyDx FFPE DNA Kit (AmoyDx, Xiamen, China), according to manufacturer’s instructions. The kit is optimized to isolate RNA molecules that are longer than 18 nucleotides from FFPE tissue samples, and to reverse as much formaldehyde modification as possible without further RNA degradation. The concentrations of the RNA samples were determined by OD260 by using a NanoDrop ND-1000 instrument. The integrity of RNA was assessed by electrophoresis on a denaturing agarose gel.

**Liquid Chromatography-Tandem Mass Spectrometry (LC-MS/MS) Acquisition**

LC-MS/MS acquisitions were performed on an Orbitrap Fusion mass spectrometer (Thermo Fisher Scientific, USA) coupled with a nanoLC (Dionex Ultimate 3000, Thermo Fisher Scientific, USA). For each sample, 1 µg dissolved peptides were delivered to an analytical column (Dikma, inspire C18, 3 µm, Canada, 150 mm×75 µm, self-packed). At a flow rate of 0.3 µl/minute, Buffer A (0.1% formic acid in H2O) and Buffer B (0.1% formic acid in 80% ACN) were used to run a gradient from 3% to 7% Buffer B for 5 min, 7% to 22% for 50 min, 22% to 35% for 12 min, 35% to 80% for 1 min, and finally 80% for 8 min.

In order to generate a spectral library, the Orbitrap Fusion mass spectrometer was operated in Data-Dependent Acquisitions (DDA) mode in our study. Full MS survey scans were acquired in the Orbitrap at 60,000 resolution at scan range of 350-1550 m/z, followed by MS/MS scans (resolution 30,000) recorded for maximum 3 seconds by higher energy collision induced dissociation (HCD, target value of 10,000, max 35 ms accumulation time) at a normalized collision energy of 30% in the Orbitrap. To maximize the number of precursors targeted for analysis, dynamic exclusion was enabled with one repeat count in 60 s exclusion time. For DIA analysis, MS/MS scans (resolution 30,000) were acquired at a range of 400-1000 m/z with an isolation window of 21 m/z (1 m/z overlapped between each window). The total cycle time was around 3 s and the rest parameters were identical to the DDA mode.

Data Processing and Analysis

The spectral library was generated by combining DDA raw data and searching against Uniprot Human Protein Database (2018.01.04) using Proteome Discoverer version 1.4 (Thermo Fisher Scientific). Carbamidomethylation on cysteine residues was set as fixed modification and methionine oxidation as variable modification. The mass tolerances were set to 10 ppm for the precursor ions and 0.02 Da for the fragments. All peptides were filtered at high confidence level. The 12 DIA raw data were converted in HTRMS Converter (Biognosys, Switzerland) and then imported intoSpectronaut (Biognosys, Switzerland) software with default settings. Peptide fragment ion peak areas were extracted by searching against above-mentioned library. The false discovery rate (FDR) was estimated with the mProphet approach and set to 1% at peptide precursor level and at 1% at protein level. Identified peptides with sequences containing miss-cleavages and methionine were excluded.Raw quantification data were log2 transformed and then normalized so that the mean was equal for each profile. T-tests analysis was performed to compared their capacity of identifying differentially expressed proteins.

**Statistical analysis**

Patient characteristics were summarized using descriptive statistics, which included frequency counts, median, and interquartile range. Categorical variables were compared using Fisher’s exact test or the Chi-quared test and continuous variables using a nonparameter T-test. We utilized the "glmnet" package to fit the logistic elastic net regression analysis [^8^](#_ENREF_8)^;^[^9^](#_ENREF_9). Our analysis was performed based on the log2 scale of the circRNA expression data. We utilized ten-fold cross-validation to select the penalty term. ROC curve was used to determine the optimal cutoff value of the interesting predictor. Progression free survival (PFS) was defined as the time from disease diagnosis until the first documented day of progression, relapse or death from any cause. Overall survival (OS) was defined as time from the date of diagnosis until death due to any cause or the last follow-up. The treatment response was assessed using computed tomography (CT) and/or positron emission tomography (PET-CT) scans. Response was defined according to the Revised Response Criteria for Malignant Lymphoma[^10^](#_ENREF_10). The log-rank test in the Kaplan-Meier survival model was used to evaluate the prognostic impact of categorical variables. Univariate and multivariate analyses with a Cox proportional hazards models and logistic regression were performed to assess significant predictors. The proportional-hazards assumption was checked for each variable before fitting Cox models. All statistical analyses were conducted with R statistic packages, version 3.6.1 (www.r-project.org). The two-sided level of significance was set at p-value < 0.05.


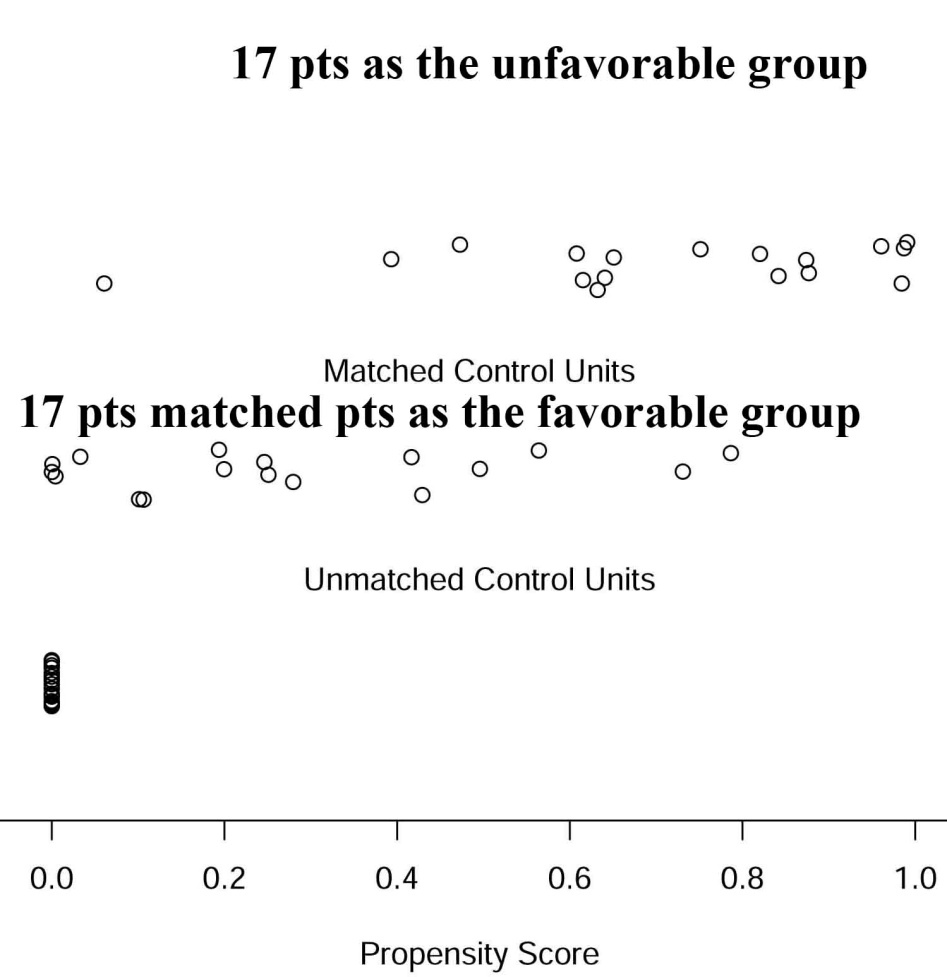


Figure S1. Propensity score analysis was used to select DLBCL patients with non-POD24.


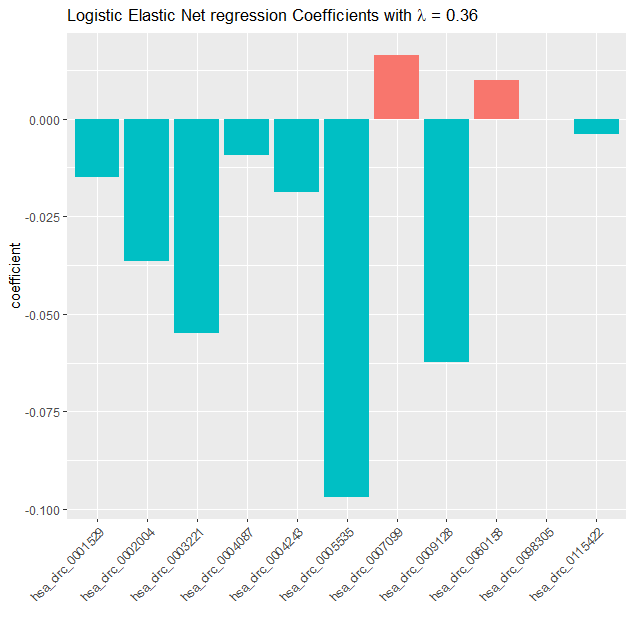
Figure S2. Differently expressed circRNAs related to POD24 by the logistic elastic net regression analysis.


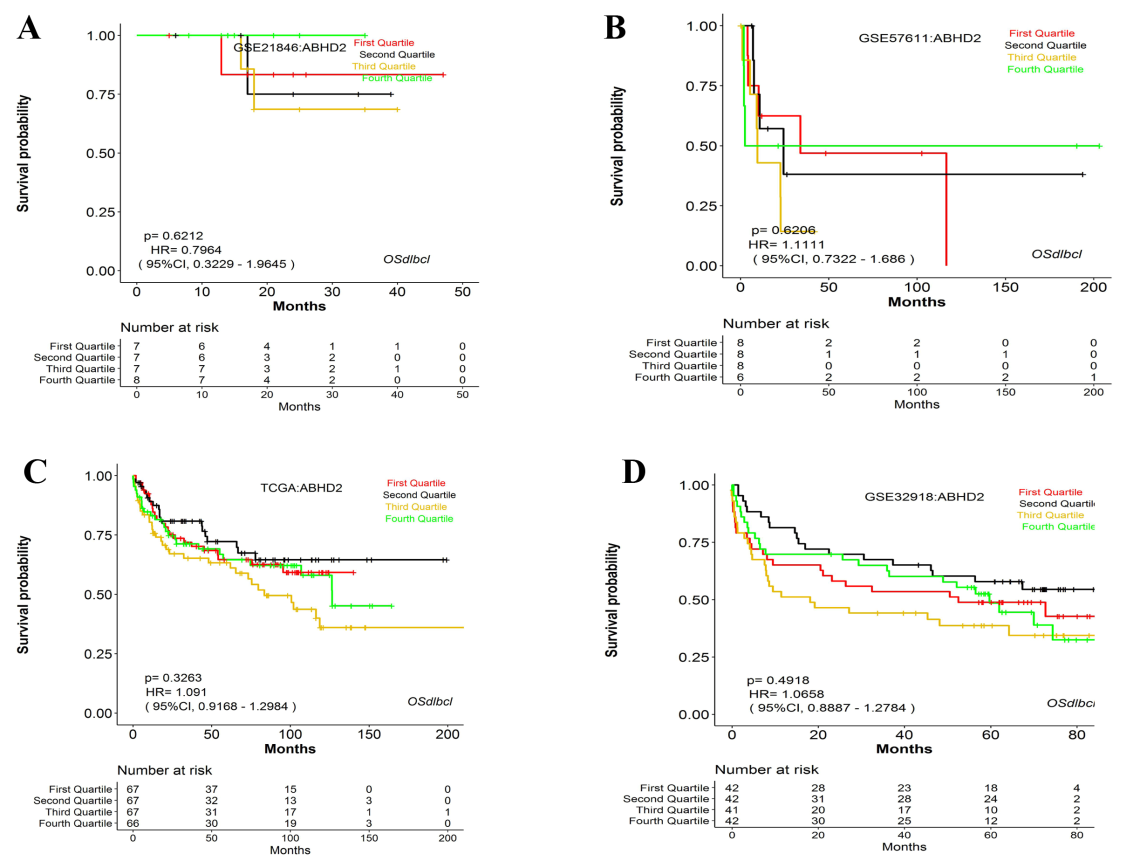


Figure S3. *ABHD2* expressions were not associated with overall survival in the published dataset. The data were analyzed from the web site (<http://bioinfo.henu.edu.cn/DLBCLCombined#collapseOne>)


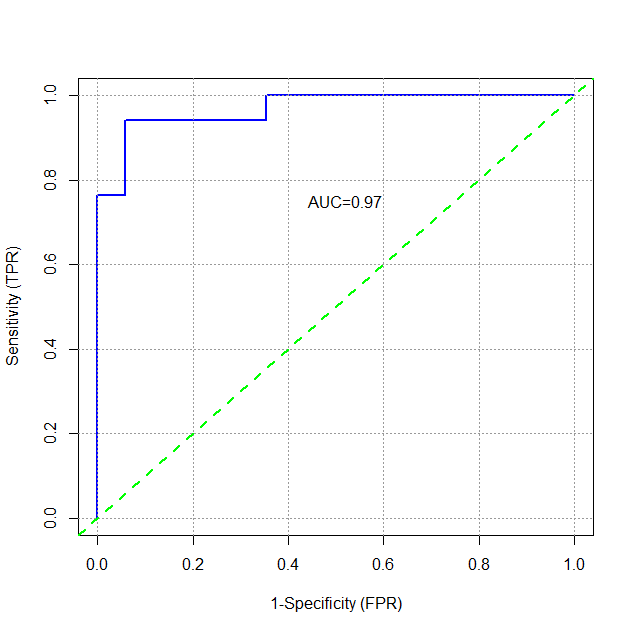


Figure S4. Receiver operating characteristic (ROC) curves of hsa_circ_0007099 expression for predicting POD24 in the 34 DLBCL patients


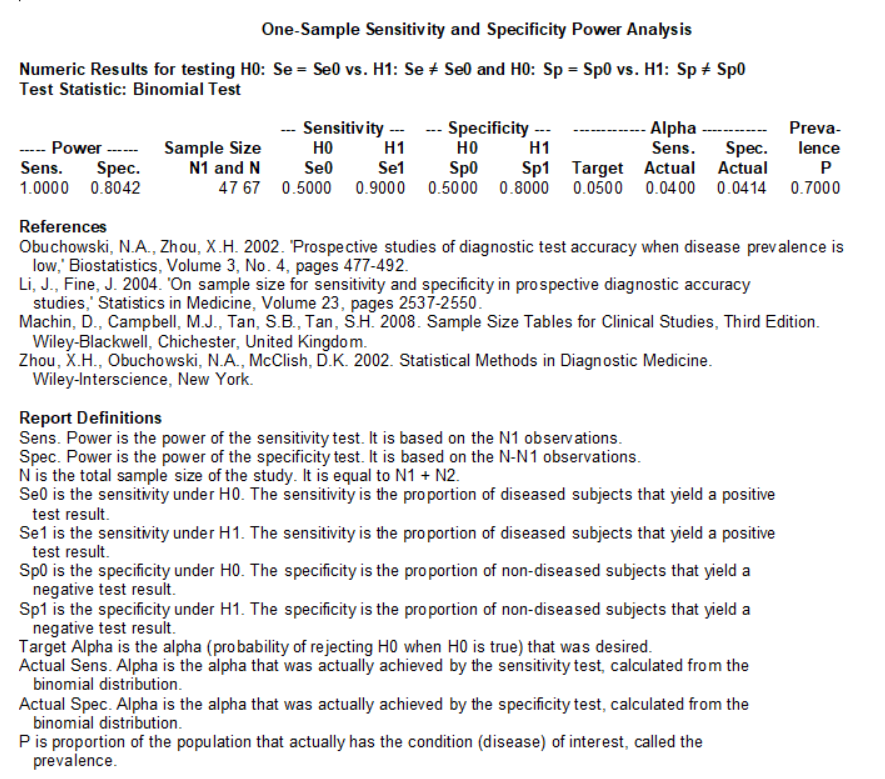


Figure S5. A total sample size of 67 (which includes 47(70%) subjects with Non-POD24) achieves 100% power to detect a change in sensitivity from 0.5 to 0.9 and 80% power to detect a change in specificity from 0.5 to 0.8 using a two-sided binomial test. The target significance level is 0.05. The actual significance level achieved by the sensitivity as well as specificity test is 0.04. Also, we enrolled 15(more than 20%) cases as lost to follow-up. Thus, we needed 82 patients in the validation set.


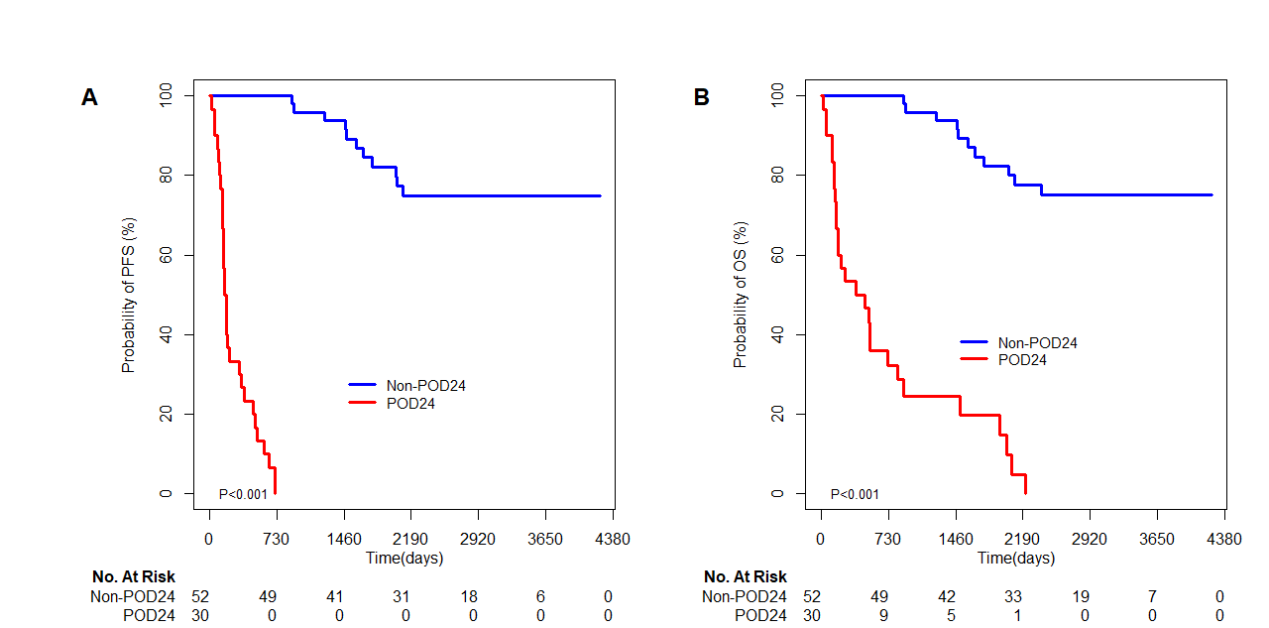


Figure S6. PFS (A) and OS (B) KM curves of patients with and without POD24 in the validation set


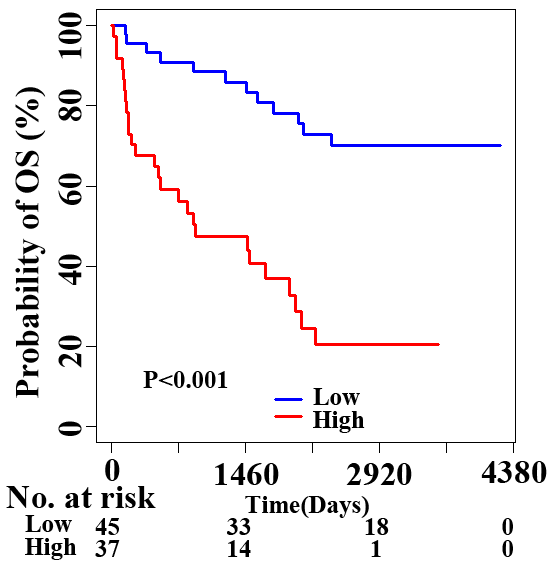


Figure S7. Survival curves of DLBCL patients for OS by high and low expressed groups


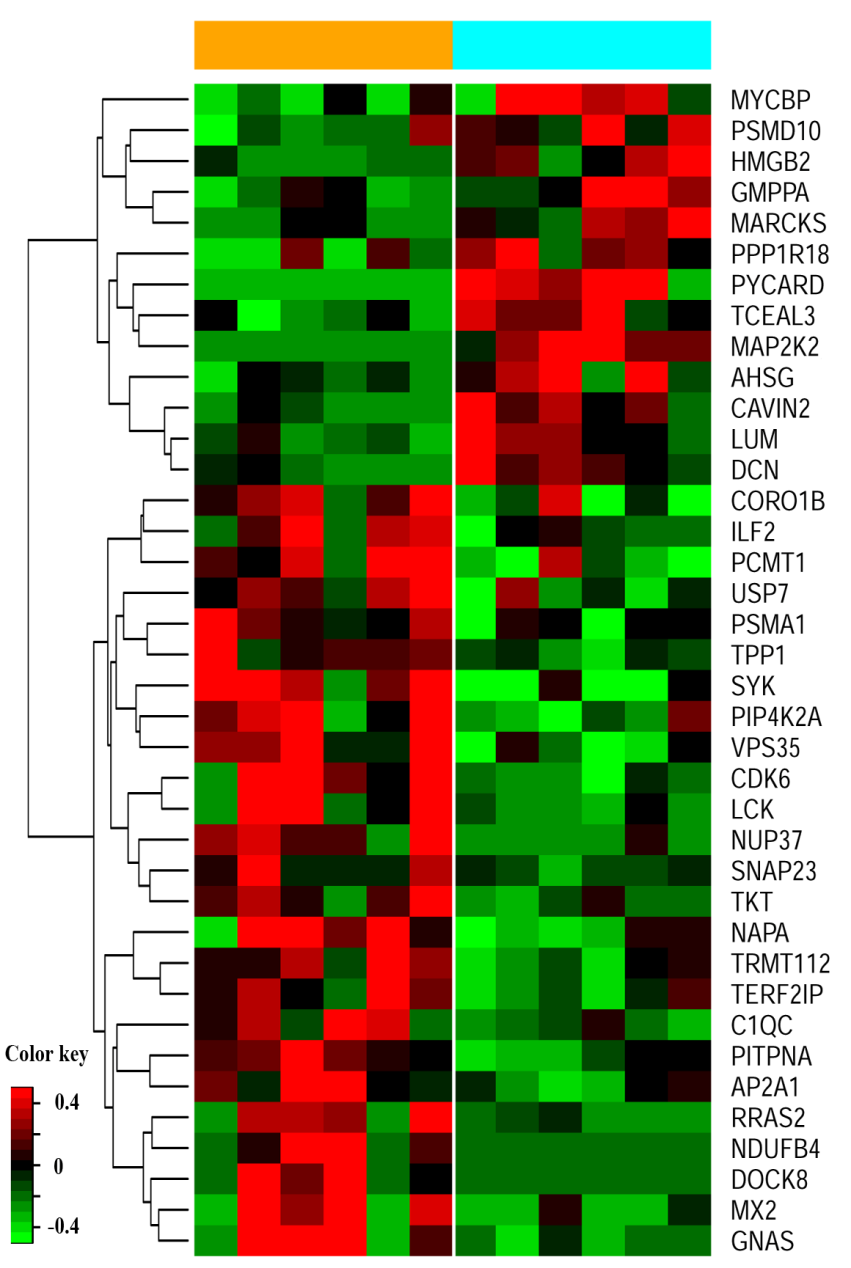


Figure S8 Heat map illustrates the differently expressed proteins between high and low groups


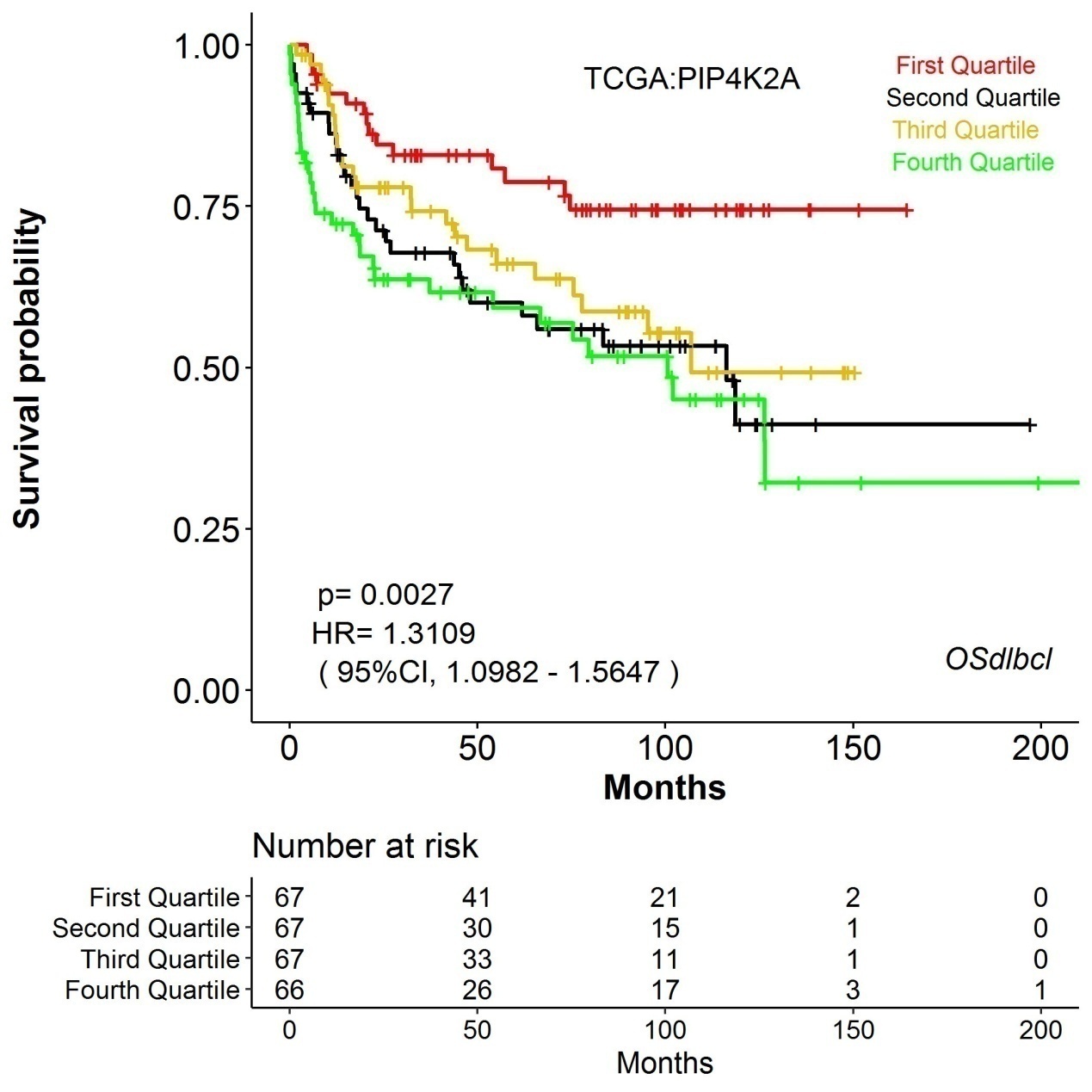


Figure S9. KM survival curves of PIP4K2A expression in DLBCL patients. This result was analyzed by the online tool (http://bioinfo.henu.edu.cn/DLBCLCombined).


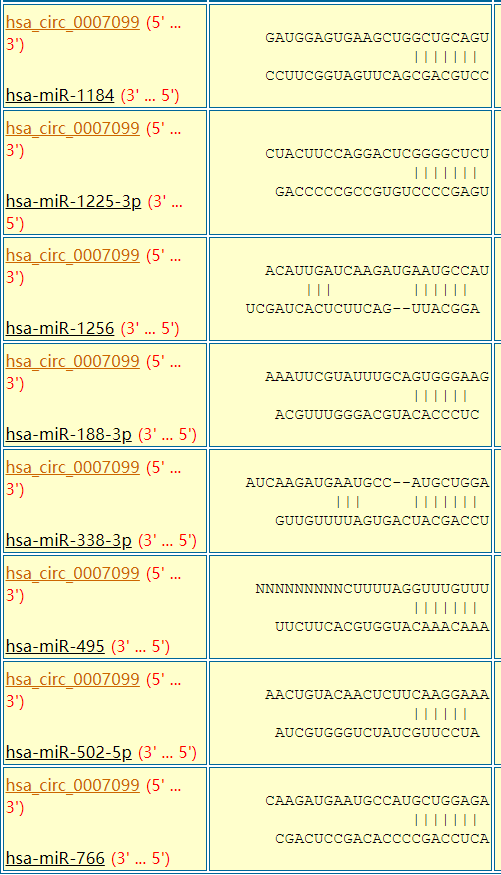


Figure S10. Seven miRNAs that potentially interacted with hsa_circ_0007099 in the CircInteractome database


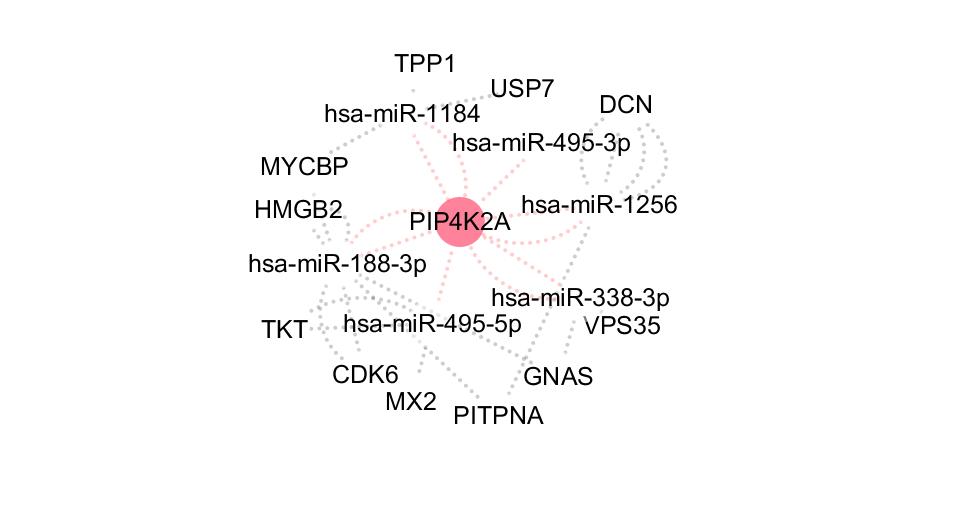


Figure S11. The miRNA-mRNA network of the genes encoding aberrantly expressed proteins and hsa_circ_0007099 sponging microRNAs.


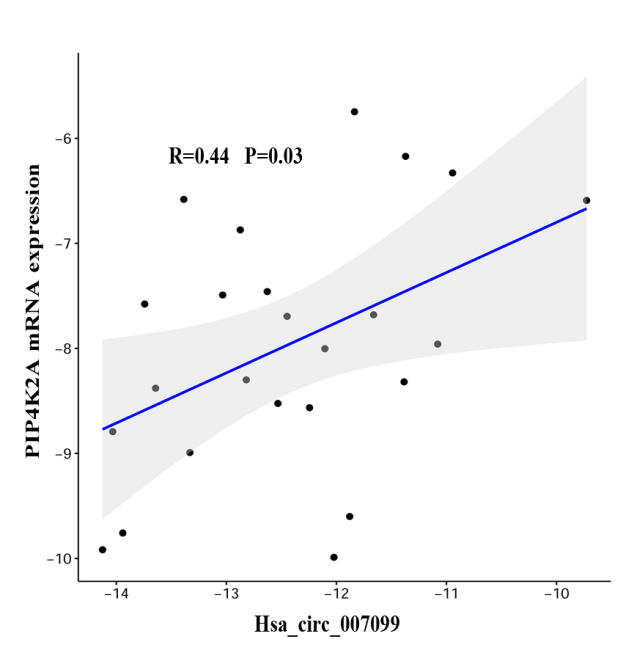


Figure S12. Has_circ_0007099 expressions were positively correlated with mRNA expressions of *PIP4K2A* in DLBCL patients.


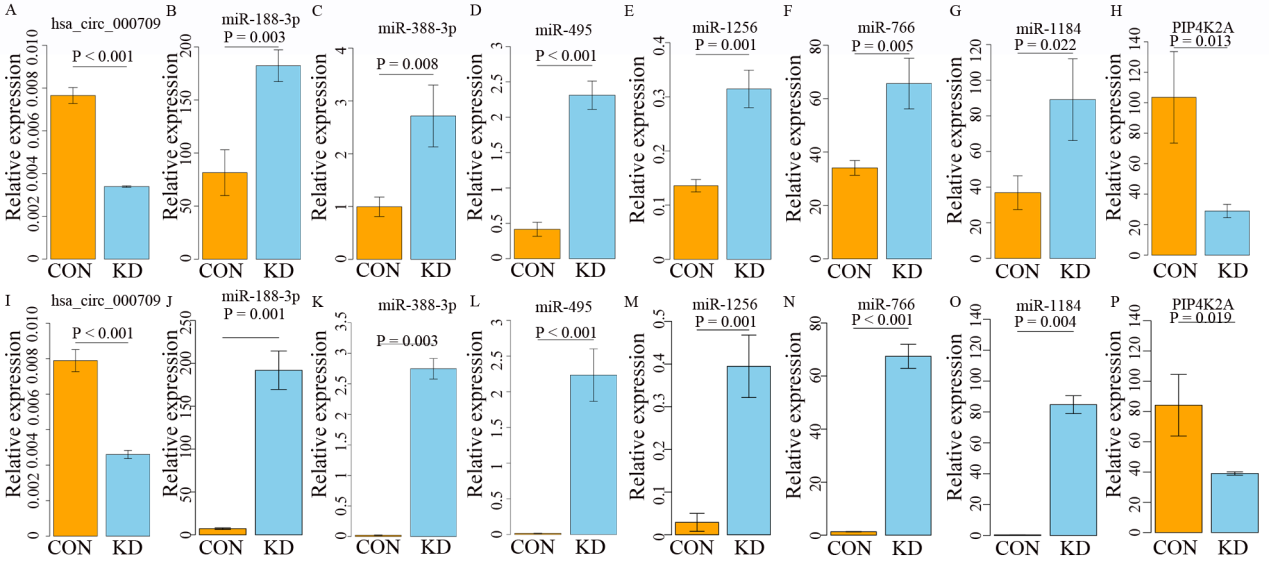


Figure S13. In vitro cellular experiments were used to validate the regulatory network. The knockdown of hsa_circ_0007099 expression significantly increased expression of miR-188-3p, miR-1256, miR-1184, miR-338-3p, miR-495-3p, miR-495-5p genes. In parallel, knockdown hsa_circ_0007099 expression significantly reduced expression of *PIP4K2A* in OCI-Ly1(A-H) and OCI-Ly10 (I-P) cell lines.

Table S1. Clinical characteristics of DLBCL patients in training and validation sets

| Variable | Training Set | | | Validation Set | | |
| --- | --- | --- | --- | --- | --- | --- |
|  | Non-POD24 | POD24 | P value | Non-POD24 | POD24 | P value |
| Number | 17 | 17 |  | 52 | 30 |  |
| Male,sex,n(%) | 8(47.1) | 11(64.7) | 0.491 | 24(46.2) | 21(70.0) | 0.042 |
| Age,Median(IQR)1 | 64.00[54.00,65.00] | 59.00[55.00,65.00] | 0.691 | 56.00[49.75,59.00] | 57.50[51.00,65.25] | 0.142 |
| Age(>60yrs),n(%) | 12(70.6) | 8(47.1) | 0.296 | 12(23.1) | 13(43.3) | 0.081 |
| ECOGPS(>=2),n(%)2 | 6(35.3) | 9(52.9) | 0.491 | 16(30.8) | 14(46.7) | 0.163 |
| StageIII/IV | 9(52.9) | 12(70.6) | 0.481 | 32(61.5) | 23(76.7) | 0.223 |
| LDH,IU/L,Median(IQR) | 254.00[212.00,319.00] | 289.00[216.00,490.00] | 0.335 | 198.00[168.50,271.00] | 356.00[247.00,490.00] | 0.007 |
| Extranodal disease, n(%) | 10(58.8) | 11(64.7) | 1 | 12(23.1) | 16(53.3) | 0.008 |
| IPI,n(%)3 |  |  | 0.898 |  |  | 0.004 |
| Low | 5(29.4) | 7(41.2) |  | 22(42.3) | 6(20.0) |  |
| Intermediate | 9(52.9) | 7(41.2) |  | 26(50.0) | 13(43.3) |  |
| High | 3(17.6) | 3(17.6) |  | 4(7.7) | 11(36.7) |  |
| Non-GCB,n(%)4 | 13(76.5) | 12(70.6) | 1 | 37(71.2) | 27(90.0) | 0.056 |
| CD10 positive | 1(5.9) | 4(23.5) | 0.335 | 9(17.3) | 4(13.3) | 0.76 |
| BCL6 positive | 15(88.2) | 15(88.2) | 1 | 36(69.2) | 18(60.0) | 0.471 |
| MUM1 positive | 13(76.5) | 15(88.2) | 0.656 | 38(73.1) | 24(80.0) | 0.597 |
| DEL,n(%)^5^ | 4(23.5) | 9(52.9) | 0.157 | 13(25.0) | 16(53.3) | 0.016 |
| B symptoms,n(%)6 | 6(35.3) | 3(17.6) | 0.438 | 0.33(0.47) | 0.53(0.51) | 0.068 |
| CR,n(%)^7^ | 17(100.0) | 3(17.6) | <0.001 | 52(100.0) | 10(33.3) | <0.001 |

^1^IQR, interquartile range; ^2^ ECOG PS, Eastern Cooperative Oncology Group performance status; ^3^IPI, International Prognostic Index;^4^ Non-GCB, Non-germinal center B-cell-like lymphoma; ^5^DEL, double expresser lymphoma; ^6^B symptoms refer to systemic symptoms of fever, night sweats, and weight loss; ^7^CR, complete remission; POD24, progression or relapse within 24 months.

Table S2. Clinical characteristics of patients with high and low hsa_circ_0007099

| Variables | Low | High |  |
| --- | --- | --- | --- |
| Number | 45 | 37 |  |
| Male,sex,n(%) | 19(42.2) | 26(70.3) | 0.015 |
| Age,Median(IQR)1 | 56.00[51.00,60.00] | 57.00[50.00,63.00] | 0.702 |
| Age (>60yrs),n(%) | 12(26.7) | 13(35.1) | 0.474 |
| ECOG PS(>=2),n(%)2 | 14(31.1) | 16(43.2) | 0.357 |
| Stage III/IV | 31(68.9) | 24(64.9) | 0.814 |
| LDH,IU/L,Median(IQR) | 201.00[169.00,321.00] | 269.00[183.50,371.50] | 0.311 |
| Extranodal disease,n(%) | 13(28.9) | 15(40.5) | 0.350 |
| IPI,n(%)3 |  |  | 0.182 |
| Low | 19(42.2) | 9(24.3) | |
| Intermediate | 20(44.4) | 19(51.4) | |
| High | 6(13.3) | 9(24.3) | |
| Non-GCB,n(%)4 | 33(73.3) | 31(83.8) | 0.294 |
| CD10 positive | 33(73.3) | 26(72.2) | 1 |
| BCL6 positive | 26(57.8) | 21(58.3) | 1 |
| MUM1 positive | 17(37.8) | 20(55.6) | 0.123 |
| DEL,n(%)^5^ | 13(28.9) | 16(43.2) | 0.246 |
| B symptoms,n(%)6 | 0.36(0.48) | 0.46(0.51) | 0.346 |
| CR,n(%)^7^ | 43(95.6) | 19(51.4) | <0.001 |

^1^IQR, interquartile range; ^2^ ECOG PS, Eastern Cooperative Oncology Group performance status; ^3^IPI, International Prognostic Index;^4^ Non-GCB, Non-germinal center B-cell-like lymphoma; ^5^DEL, double expresser lymphoma; ^6^B symptoms refer to systemic symptoms of fever, night sweats, and weight loss; ^7^CR, complete remission; POD24, progression or relapse within 24 months.

Table S3. Univariate analyses of PFS and OS in DLBCL patients

| Variables | Progression free survival | | Overall survival | |
| --- | --- | --- | --- | --- |
|  | Pvalue | HR(95%CI) | Pvalue | HR(95%CI) |
| CircRNA (High vs.Low) | <0.001 | 4.903(2.498,9.623) | <0.001 | 4.796(2.373,9.694) |
| CircRNA (log2 transformed) | 0.001 | 1.254(1.095,1.435) | 0.001 | 1.297(1.111,1.514) |
| IPI |  |  |  |  |
| Intermediate vs. Low | 0.326 | 1.494(0.671,3.325) | 0.304 | 1.554(0.671,3.602) |
| High vs. Low | <0.001 | 5.26(2.241,12.345) | <0.001 | 6.601(2.676,16.283) |
| Sex (Male vs. Female) | 0.097 | 1.702(0.908,3.191) | 0.064 | 1.865(0.964,3.61) |
| DEL | 0.073 | 1.761(0.949,3.266) | 0.117 | 1.675(0.878,3.192) |
| Hans classification | 0.187 | 1.73(0.766,3.909) | 0.143 | 1.919(0.801,4.597) |

IPI, International Prognostic Index; Non-GCB, Non-germinal center B-cell-like lymphoma; DEL, double expresser lymphoma, Hans classification includes GCB and Non-GCB subtypes.

Table S4. Multivariate analyses of PFS and OS in DLBCL patients

| Variables | Progression free survival | | Overall survival | |
| --- | --- | --- | --- | --- |
|  | Pvalue | HR(95%CI) | P value | HR(95%CI) |
| Hsa_circ_0007099  (log2 transformed) | 0.036 | 1.16(1.01,1.333) | 0.036 | 1.18(1.011,1.378) |
| IPI |  |  |  |  |
| Intermediate vs. Low | 0.396 | 1.427(0.628,3.245) | 0.396 | 1.454(0.613,3.451) |
| High vs. Low | 0.001 | 4.861(1.97,11.997) | <0.001 | 6.83(2.542,18.352) |
| Sex (Male vs. Female) | 0.136 | 1.663(0.852,3.247) | 0.093 | 1.813(0.905,3.633) |
| DEL | 0.208 | 1.516(0.793,2.899) | 0.373 | 1.363(0.69,2.692) |
| COO classification | 0.207 | 1.745(0.734,4.146) | 0.123 | 2.094(0.819,5.352) |

IPI, International Prognostic Index; Non-GCB, Non-germinal center B-cell-like lymphoma; DEL, double expresser lymphoma

Table S5. Multivariate analyses of PFS and OS in DLBCL patients

| Variables | Progression free survival | | Overall survival | |
| --- | --- | --- | --- | --- |
|  | Pvalue | HR(95%CI) | P value | HR(95%CI) |
| CircRNA (High vs. Low) | <0.001 | 3.968(1.903,8.275) | 0.001 | 3.836(1.779,8.275) |
| IPI |  |  |  |  |
| Intermediate vs. Low | 0.884 | 1.064(0.46,2.461) | 0.814 | 1.111(0.461,2.682) |
| High vs. Low | 0.006 | 3.64(1.452,9.126) | 0.001 | 5.487(2.031,14.821) |
| Sex (Male vs. Female) | 0.288 | 1.435(0.737,2.792) | 0.227 | 1.54(0.764,3.105) |
| DEL | 0.215 | 1.506(0.788,2.879) | 0.414 | 1.33(0.671,2.635) |
| Hans classification | 0.138 | 1.907(0.813,4.476) | 0.084 | 2.276(0.896,5.779) |

IPI, International Prognostic Index; Non-GCB, Non-germinal center B-cell-like lymphoma; DEL, double expresser lymphoma，Hans classification includes Non-GCB and GCB subtypes.

Table S6. Clinical characteristics of high and low hsa_circ_0007099 expressers for protein profiling analyses

| Variables | Low | High |  |
| --- | --- | --- | --- |
| Number | 6 | 6 |  |
| Male,sex,n(%) | 1 ( 16.7) | 5 ( 83.3) | 0.08 |
| Age,Median(IQR)1 | 62.00 [55.75, 74.25] | 59.50 [56.25, 63.50] | 0.749 |
| Age (>60yrs),n(%) | 4 ( 66.7) | 3 ( 50.0) | 1 |
| ECOG PS(>=2),n(%)2 | 4 ( 66.7) | 6 (100.0) | 0.455 |
| Stage III/IV | 3 ( 50.0) | 4 ( 66.7) | 1 |
| LDH,IU/L,Median(IQR) | 318.00 [238.75, 851.75] | 490.00 [380.50, 694.75] | 0.521 |
| Extranodal disease,n(%) | 5 ( 83.3) | 4 ( 66.7) | 1 |
| IPI,n(%)3 |  |  | 1 |
| Low | 0 ( 0.0) | 0 ( 0.0) | |
| Intermediate | 3 ( 50.0) | 2 ( 33.3) | |
| High | 3 ( 50.0) | 4 ( 66.7) | |
| Non-GCB,n(%)4 | 2 ( 33.3) | 5 ( 83.3) | 0.242 |
| CD10 positive | 1 ( 16.7) | 1 ( 16.7) | 1 |
| BCL6 positive | 4 ( 66.7) | 5 ( 83.3) | 1 |
| MUM1 positive | 2 ( 33.3) | 6 (100.0) | 0.061 |
| DEL,n(%)^5^ | 1 ( 16.7) | 3 ( 50.0) | 0.545 |
| B symptoms,n(%)6 | 3 ( 50.0) | 1 ( 16.7) | 0.545 |
| CR,n(%)^7^ | 6 (100.0) | 3(50.0) | 0.018 |

^1^IQR, interquartile range; ^2^ ECOG PS, Eastern Cooperative Oncology Group performance status; ^3^IPI, International Prognostic Index;^4^ Non-GCB, Non-germinal center B-cell-like lymphoma; ^5^DEL, double expresser lymphoma; ^6^B symptoms refer to systemic symptoms of fever, night sweats, and weight loss; ^7^CR, complete remission.

Table S7. Reactome Pathways analyses in the differently expressed proteins

| Names of pathways | P value | Hit Count in Query List | Hit Count in Genome | Hit in Query List | |
| --- | --- | --- | --- | --- | --- |
| Host Interactions of HIV factors | 0.00002705 | 5 | 136 | PSMD10,LCK,AP2A1,NUP37,PSMA1 | |
| CLEC7A (Dectin-1) signaling | 0.0001506 | 4 | 103 | SYK,PSMD10,PSMA1,PYCARD | |
| Nef Mediated CD4 Down-regulation | 0.0003014 | 2 | 10 | LCK,AP2A1 | |
| DAP12 signaling | 0.0003201 | 6 | 359 | SYK,PSMD10,LCK,MAP2K2,PSMA1,PIP4K2A | |
| Signaling by the B Cell Receptor (BCR) | 0.0003633 | 5 | 236 | SYK,PSMD10,LCK,PSMA1,PIP4K2A | |
| HIV Infection | 0.0003924 | 5 | 240 | PSMD10,LCK,AP2A1,NUP37,PSMA1 | |
| Innate Immune System | 0.0003932 | 11 | 1312 | SYK,SNAP23,AHSG,C1QC,PSMD10,ILF2,LCK,MAP2K2,PSMA1,PYCARD,PIP4K2A | |
| DAP12 interactions | 0.0003984 | 6 | 374 | SYK,PSMD10,LCK,MAP2K2,PSMA1,PIP4K2A | |
| Fc epsilon receptor (FCERI) signaling | 0.0004397 | 6 | 381 | SYK,PSMD10,LCK,MAP2K2,PSMA1,PIP4K2A | |
| NGF signalling via TRKA from the plasma membrane | 0.0004976 | 6 | 390 | PSMD10,LCK,AP2A1,MAP2K2,PSMA1,PIP4K2A | |
| Infectious disease | 0.0005182 | 6 | 393 | PSMD10,LCK,AP2A1,MAP2K2,NUP37,PSMA1 | |
| C-type lectin receptors (CLRs) | 0.0005856 | 4 | 147 | SYK,PSMD10,PSMA1,PYCARD | |
| Interleukin-2 signaling | 0.0006166 | 5 | 265 | SYK,PSMD10,LCK,MAP2K2,PSMA1 | |
| Signalling by NGF | 0.001515 | 6 | 483 | PSMD10,LCK,AP2A1,MAP2K2,PSMA1,PIP4K2A | |
| Nef-mediates down modulation of cell surface receptors by recruiting them to clathrin adapters | 0.001516 | 2 | 22 | LCK,AP2A1 |  |
| ER-Phagosome pathway | 0.001542 | 3 | 87 | SNAP23,PSMD10,PSMA1 | |
| Disease | 0.001594 | 8 | 867 | PSMD10,LUM,LCK,AP2A1,DCN,MAP2K2,NUP37,PSMA1 | |

Table S8. Prediction relationship between miRNA targeted genes in silico analysis

| MicroRNAs | Symbols | start | end | number_of_pairings | binding_region_length | longest_consecutive_pairings | position |
| --- | --- | --- | --- | --- | --- | --- | --- |
| hsa-miR-188-3p | CDK6 | 10587 | 10611 | 17 | 24 | 9 | 3UTR |
| hsa-miR-188-3p | CDK6 | 10596 | 10620 | 17 | 24 | 9 | 3UTR |
| hsa-miR-1256 | DCN | 1098 | 1130 | 21 | 32 | 6 | 3UTR |
| hsa-miR-1256 | DCN | 978 | 1010 | 21 | 32 | 6 | 3UTR |
| hsa-miR-1256 | DCN | 865 | 897 | 21 | 32 | 6 | 3UTR |
| hsa-miR-1256 | DCN | 1653 | 1673 | 15 | 20 | 6 | 3UTR |
| hsa-miR-188-3p | GNAS | 1221 | 1269 | 18 | 25 | 13 | 3UTR |
| hsa-miR-338-3p | GNAS | 2506 | 2520 | 12 | 14 | 9 | 3UTR |
| hsa-miR-188-3p | HMGB2 | 766 | 791 | 14 | 25 | 9 | 3UTR |
| hsa-miR-188-3p | HMGB2 | 753 | 778 | 14 | 25 | 9 | 3UTR |
| hsa-miR-495-5p | MX2 | 3037 | 3056 | 11 | 13 | 11 | 3UTR |
| hsa-miR-1184 | MYCBP | 1443 | 1473 | 20 | 30 | 9 | 3UTR |
| hsa-miR-188-3p | MYCBP | 1782 | 1816 | 17 | 34 | 8 | 3UTR |
| hsa-miR-1184 | PIP4K2A | 3002 | 3052 | 20 | 50 | 8 | 3UTR |
| hsa-miR-1184 | PIP4K2A | 3225 | 3275 | 20 | 50 | 8 | 3UTR |
| hsa-miR-1256 | PIP4K2A | 1704 | 1726 | 18 | 22 | 7 | 3UTR |
| hsa-miR-1256 | PIP4K2A | 1481 | 1503 | 18 | 22 | 7 | 3UTR |
| hsa-miR-188-3p | PIP4K2A | 2958 | 2983 | 18 | 25 | 9 | 3UTR |
| hsa-miR-188-3p | PIP4K2A | 3181 | 3206 | 18 | 25 | 9 | 3UTR |
| hsa-miR-338-3p | PIP4K2A | 1605 | 1624 | 17 | 19 | 11 | 3UTR |
| hsa-miR-338-3p | PIP4K2A | 1382 | 1401 | 17 | 19 | 11 | 3UTR |
| hsa-miR-495-3p | PIP4K2A | 1656 | 1673 | 15 | 17 | 11 | 3UTR |
| hsa-miR-495-5p | PIP4K2A | 2835 | 2848 | 12 | 13 | 12 | 3UTR |
| hsa-miR-1256 | PITPNA | 2200 | 2223 | 18 | 23 | 10 | 3UTR |
| hsa-miR-188-3p | PITPNA | 1233 | 1260 | 17 | 21 | 13 | 3UTR |
| hsa-miR-495-5p | TKT | 2532 | 2548 | 14 | 16 | 11 | 3UTR |
| hsa-miR-495-5p | TKT | 2367 | 2383 | 14 | 16 | 11 | 3UTR |
| hsa-miR-1184 | TPP1 | 1787 | 1826 | 20 | 39 | 8 | 3UTR |
| hsa-miR-1184 | USP7 | 5694 | 5712 | 17 | 18 | 17 | 3UTR |
| hsa-miR-338-3p | VPS35 | 3618 | 3637 | 18 | 19 | 18 | 3UTR |

This result was analyzed using the online tool (http://mirwalk.umm.uni-heidelberg.de/human/gene/5305/?sort=startend)
